# Lysine Acetyltransferase TIP60 Restricts Nerve Injury by Activating IKKβ/SNAP23 Axis‐Mediated Autophagosome‐Lysosome Fusion in Alzheimer's Disease

**DOI:** 10.1111/cns.70095

**Published:** 2024-11-05

**Authors:** Wei Wang, Jun Min, Qinghua Luo, Xunhu Gu, Min Li, Xu Liu

**Affiliations:** ^1^ Department of Neurology, The 2nd Affiliated Hospital, Jiangxi Medical College Nanchang University Nanchang Jiangxi Province P.R. China; ^2^ Institute of Neuroscience Nanchang University Nanchang Jiangxi Province P.R. China; ^3^ Jiangxi Provincial Clinical Medical Research Center for Neurological Disorders Nanchang Jiangxi Province P.R. China

**Keywords:** Alzheimer's disease, autophagy, IKKβ, SNAP23, TIP60

## Abstract

**Objective:**

The hyperphosphorylation of Tau protein is considered an important cause of neuronal degeneration in Alzheimer's disease (AD). The disruption of neuronal histone acetylation homeostasis mediated by Tip60 HAT is a common early event in neurodegenerative diseases, but the deeper regulatory mechanism on β‐amyloid peptide (Aβ)‐induced neurotoxicity and autophagic function in AD is still unclear.

**Methods:**

AD models were established both in APP/PS1 mice and Aβ_1–42_‐treated SH‐SY5Y cells. The Morris water maze test (MWM) was performed to examine mouse cognitive function. Neurological damage in the hippocampus was observed by hematoxylin–eosin (H&E), Nissl's, terminal deoxynucleotidyl transferase dUTP nick end labeling (TUNEL), and NeuN staining. Autophagosome‐lysosome fusion was detected by immunohistochemistry, immunofluorescence, and Lyso‐Tracker Red staining. Cell viability and apoptosis were evaluated by CCK‐8 assay and flow cytometry. The molecular interactions were verified by co‐immunoprecipitation (Co‐IP), dual luciferase assays, and ChIP detections. The RNA and autophagy‐lysosome‐related proteins were assessed by Western blot and RT‐qPCR.

**Results:**

TIP60 overexpression improved cognitive deficits and neurological damage and restored the impairment of autophagy‐lysosomes fusion in vivo. Similarly, the upregulation of TIP60 in Aβ_1–42_‐treated SH‐SY5Y cells suppressed neuronal apoptosis and tau phosphorylation through the activating autophagy‐lysosome pathway. Mechanistically, TIP60 activated IKKβ transcription by promoting SOX4 acetylation, thus leading to the translocation of SNAP23 to STX17‐contained autophagosomes. Moreover, the protective roles of TIP60 in neuron damage were abolished by the inhibition of SOX4/IKKβ signaling.

**Conclusion:**

Collectively, our results highlighted the potential of the TIP60 target for AD and provided new insights into the mechanisms underlying neuroprotection in this disorder.

## Graphical abstract

The schematic diagram of TIP60 alleviates cognitive impairment and neurological damage in AD by activating SNAP23‐mediated autophagosome‐lysosome fusion through activating SOX4/IKKβ signaling.

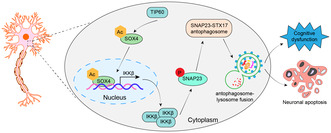

**Abbreviations**
Aβ: amyloid beta
AD: Alzheimer's disease
CCK‐8: cell counting kit‐8
ChIP: chromatin immunoprecipitation
Co‐IP: co‐immunoprecipitation
ELISA: enzyme linked immunosorbent assay
HAT: histone acetyltransferase
HE: hematoxylin‐eosin
HRP: horseradish peroxidase
IKK: IκB kinase
MUT: mutated
MWM: Morris water maze
NFT: neurofibrillary tangles
SNAP: synaptic associated protein
TUNEL: terminal deoxynucleotidyl transferase‐mediated dUTP nick‐end labeling
WHO: World Health Organization
WT: wild type

## 1 Introduction

Alzheimer's disease (AD) is a neurodegenerative disease with insidious onset. In 2015, the number of global dementia patients was 47.47 million, costing over 800 billion US dollars. With the increasing aging population, it is predicted by the World Health Organization (WHO) that this number will reach 75.63 million by 2030 and 135.46 million by 2050, creating a huge economic burden on world [1, 2]. The pathological features of AD patients include memory loss, cognitive dysfunction, tau‐containing neurofibrillary tangles, and β‐amyloid (Aβ)‐containing extracellular plaques [3, 4]. Autophagy is an important degradation pathway for clearing abnormal protein aggregates in mammalian cells, responsible for protein homeostasis and maintaining neuronal health [5]. Studies have shown that autophagy defects occur in the early stages of AD. Meanwhile, autophagy plays an important role in the production and metabolism of Aβ and the assembly of Tau, and its dysfunction may lead to the progression of AD [6, 7]. Therefore, autophagy may be a new target for developing an efficient therapeutic strategy for AD.

The changes in transcriptional and post‐transcriptional levels of epigenetics are closely related to the occurrence and development of various neurodegenerative diseases, such as AD, Huntington's disease, and Parkinson's disease [8, 9]. TIP60 is a highly conserved acetyltransferase, and the lack of TIP60 acetyltransferase activity is associated with dysregulation of various cellular pathways, including apoptosis, dNTP biosynthesis, and oxidative stress response [10]. Cognitive decline is an important indicator of the preclinical stage of AD, and histone acetylation homeostasis is crucial for mediating epigenetic genes during neural development. The disruption of neurohistone acetylation state mediated by TIP60 histone acetyltransferase (HAT) is a common early event in neurodegenerative disease [11]. Studies have shown that in a Drosophila model induced by Aβ_42_, an increase in Tip60 HAT levels in the brain can prevent AD functional lesions, including Aβ plaque accumulation, neuronal death, cognitive defects, and shortened lifespan [12]. Moreover, the increased TIP60 alleviates the balance of TIP60/HDAC2 by reducing the level of HDAC2, which reverses neurogenetic changes, activates the synaptic plasticity gene, and restores brain morphology and cognition [13]. However, the deeper regulatory mechanism is not yet clear, and further research is needed.

The IκB kinase (IKK) complex consists of two catalytic subunits, IKKα and IKKβ, and a regulatory subunit, IKKγ, which are responsible for regulating the activation of NF‐κB and are closely related to inflammatory response [14, 15]. Neuroinflammation plays an important role in promoting neuronal pathology and cell death in many types of neurodegenerative diseases, and activated IKKβ triggers the NF‐κB pathway to express immune and inflammatory‐related genes in AD mice [16]. Our previous study has shown that constitutively overexpressed IKKβ can activate autophagy and repress RIPK1‐induced necroptosis in Aβ‐induced SH‐5Y5Y cells [17]. Besides, activation of IKKβ reduces the accumulation of phosphorylated Tau protein in AD's disease model by inducing autophagy, thereby resulting in delayed behavior in mice [18]. A previous study has revealed that IKKβ mediates phosphorylation of synaptic associated protein (SNAP) 23 at the Ser95 and Ser120 sites in rats and the Ser120 sites in humans, which leads to the transfer of SNAP23 from the plasma membrane to autophagosomes, thereby regulating the STX17‐SNARE complex‐mediated autolysosome pathway and ultimately leading to pancreatic acinar injury [19]. The autophagy process involves membrane fusion, and membrane fusion‐mediated cell membrane substance transport requires the interaction of soluble N‐ethylmaleimide‐sensitive fusion protein attachment protein receptor (SNARE) family members to form SNARE complex [20]. The members of the SNAP subfamily include SNAP25, SNAP23, SNAP47, and SNAP29, which interact with other SNARE to form SNARE complexes and participate in various processes of autophagy [21]. Therefore, we speculated that SNAP23 autophagosome formation catalyzed by IKKβ may be an important pathway for clearing p‐Tau.

In the present study, we investigated the function and underlying mechanisms of acetyltransferase TIP60 relating to AD. Gain‐of‐function of TIP60 improved cognitive ability, decreased neurological damage, and restored the impairment of the autophagy‐lysosomes pathway in APP/PS1 transgenic mice. In cellular level, TIP60 was downregulated in an AD cell model, and the upregulation of TIP60 alleviated neuron injury by activating IKKβ/SNAP23‐mediated autophagy‐lysosome fusion. The relevant research will provide a theoretical basis for the systematic exploration of key molecules and their regulatory mechanisms in the AD process and for the clinical search for AD drug targets.

## 2 Materials and Methods

### 2.1 Construction of AD Model

Eight‐month‐old APP‐PSEN1 ΔE9 transgenic AD mice (APP/PS1) and wild‐type (WT) NonTg littermates from Shanghai Model Organisms (Shanghai, China) were used in the study, which complies with the Guide for the Animal Care and Use Committee of The Second Affiliated Hospital of Nanchang University. All experimental procedures were approved by The Second Affiliated Hospital of Nanchang University (Review [2022] No. (075)). AD mouse model APP/PS1 transgenic mice display classical pathological characteristics including cognitive deficits, Aβ deposition, and tau pathology. All mice were bred in filer‐top cages and maintained on a 12‐h light/dark cycle, with free access to food and water. Mice were randomly divided into four groups (*n* = 12): control, AD, AD+AAV‐NC, and AD‐AAV‐TIP60. Adeno‐associated virus (AAV) carrying the TIP60 gene (AAV‐TIP60, 1 × 10^10^ pfu/mouse) or NC oligonucleotides were microinjected into the hippocampus tissues with a stereotaxic apparatus using the following coordinates: AP (anterior posterior), −0.5 mm; ML (mediolateral), 1.00 mm; and DV (dorsoventral), −3.0 mm. The injection frequency of AAV was performed once a week for a period of 2 months. One week after AAV injection, the mice were euthanized and hippocampal tissue was taken for subsequent testing.

### 2.2 Morris Water Maze Test

A Morris Water Maze (MWM) test was performed after 2 months of intrahippocampal injection. Throughout the entire experimental process, MWM was placed in a dark environment, separating the mice's time limit from the operator to ensure that the extrauterine reference remained unchanged. Spatial learning and memory ability were evaluated within 24 h after the 6 consecutive days' training. The escape latency, the number of crossing the platforms, and the time spent in the target quadrant (TQ) were automatically recorded in each group.

### 2.3 RNA Exaction and Real‐Time Quantitative PCR Assay

Total RNA was extracted from SH‐SY5Y cells using the Direct‐zol RNA MiniPrep kit (ZYMO research), and brain tissue samples were homogenized via Trizol reagent (Life Technologies, Gaithersburg, MD, USA). RNA was then reverse transcribed with a High‐Capacity cDNA Reverse Transcription Kit (Thermo Fisher Scientific, Waltham, MA, USA). The real‐time quantitative PCR (RT‐qPCR) assay was performed using SYBR Green PCR Master Mix (Thermo Fisher Scientific) on the ABI‐7500 system (Applied Biosystems, USA). Each sample was set in triplicate. The relative expression levels of the tested mRNA relative to β‐actin were counted by the 2^−∆∆CT^ formula. The sequence information for RT‐qPCR was provided in Table 1.

**TABLE 1:** The sequences of RT‐qPCR primer enrolled in the present experiment.

Gene	Forward (5′–3′)	Reverse (5′–3′)
TIP60 (M)	GTGTCTGCACTTCACTCCCA	TGTTCTTCGGCCTGTCTCAC
TIP60 (H)	GAAGATGGCGGAGGTGGTG	CCACTGATGTCCTTCACGCT
LAMP1 (M)	CACAGGGTCAACCTCTGGAC	GATGCCCAGTGTACAACCCA
LAMP1 (H)	CTGGTAACGCCGCTGTCTCT	TAAACATTGCTGCTGACGCAC
CTSB (M)	CACTGTCCTTACCTGGCCTC	CCACAGACCCAGAAGTGACC
CTSB (H)	CTCCTGCTGGCTGTAATGGT	CCAGGCTCACAGATCTTGCT
CTSF (M)	CCTTGCAATGATCCCCTGGT	CCTTGGCATTCACTGGGCTA
CTSF (H)	TCGCCCCGCTGGAGG	CCCCAGGCCTGAAAGCTG
SNAP25 (H)	ACCAGTTGGCTGATGAGTCG	ACACGATCGAGTTGTTCTCCT
SNAP23 (H)	CCTCGCCCGCTTGAGTTTT	AGACACAAAGGCCACAGCAT
SNAP29 (H)	CAAGTGCTGCAACAGTGCAT	CAGAATCCCTGAGGTGGCTG
SNAP47 (H)	CACAGGGTCCCAGAAGCAAGG	TTCTGCCTCTTCAAATCCGGC
IKKβ (H)	GCTGCAACTGATGCTGATGT	TGTCACAGGGTAGGTGTGGA
SOX4 (H)	TCCTCCTCTTCCTCCTCCTC	TAGTCCGGGAACTCGAAGTG
GAPDH (H)	CCAGGTGGTCTCCTCTGA	GCTGTAGCCAAATCGTTGT
GAPDH (M)	AGCCCAAGATGCCCTTCAGT	CCGTGTTCCTACCCCCAATG

### 2.4 HE Staining

HE staining was used to analyze the histopathology of hippocampal tissues. Mouse hippocampus were isolated and fixed with 10% formaldehyde solution, embedded in paraffin, sectioned at 4 μm thickness, and stained with HE. The sections were finally collected onto slides, and the neurons in the hippocampal tissues were observed using the Cytation 5 Cell Imaging Multi‐Mode Reader (Biotek, Vermont, USA).

### 2.5 Nissl's Staining

Nissl's staining was applied to detect the histopathology changes of hippocampal neurons. In brief, the hippocampal sections with a thickness of 4 μm were immersed in 0.1% toluidine blue (Solarbio), followed by 70% alcohol and 95% alcohol, and dehydrated in absolute alcohol successively. Finally, the sections were stained with Nissl staining solution (Solarbio) for 5 min. Total numbers of neurons per view were counted by Image J software.

### 2.6 TUNEL Staining

Hippocampal neuronal apoptosis was assessed by TUNEL containing streptavidin‐Fluor 594 (Thermo Fisher Scientific) and then incubated with DAPI to counterstain the nucleus. The apoptosis rate (%) in each group was calculated as apoptosis cell number (red)/total cell number (blue) × 100%.

### 2.7 Western Blot

The whole cell lysates from SH‐SY5Y cells or hippocampal tissue samples were homogenized in RIPA buffer with protease inhibitor cocktail using TissueLyzer (QIAGEN, Duesseldorf, Germany). The equal protein loading (50 μg) was separated by 12% SDS‐PAGE and then transferred onto polyvinylidene fluoride (PVDF) membranes (Bio‐Rad, Hercules, CA, USA). The membranes were probed with the following primary antibodies against p‐Tau (Ser 396, sc‐101815, Santa Cruz, CA, USA), Tau (sc‐390476, Santa Cruz), LC3B (#83506, Cell Signaling Technology, Danvers, MA, USA), p62 (#39749, Cell Signaling Technology), lysosomal‐associated membrane protein‐1 (LAMP1, ab62562, Abcam, Cambridge, MA, USA), IKKβ (#8943), SNAP23 (ab229085, Abcam), STX17 (#31261, Cell Signaling Technology), SOX4 (PA5‐41442, Thermo Fisher Scientific), and together with GAPDH (#2118, Cell Signaling Technology) as the internal control. Protein bands were visualized by enhanced chemiluminescent reaction (Millipore) and quantified for density by ImageJ software.

### 2.8 Measurement of Aβ40 and Aβ42 Contents

The levels of Aβ40 and Aβ42 in hippocampus tissues were determined by an ELISA assay kit. The tissue samples were homogenized in 5 volumes of CHAPS cell extract buffer (Cell Signaling Technology) containing a 1% protease inhibitor (Sigma‐Aldrich, USA). After centrifugation, the supernatants were harvested for determining Aβ40 and Aβ42 by enzyme‐linked immunosorbent assay (ELISA) kits (Invitrogen, USA) and performed as described in manufacturer protocols. The OD 450 nm value was measured with a microplate spectrophotometer (Bio‐Tek).

### 2.9 Preparation of Aβ Oligomer

The preparation of the soluble Aβ oligomer was conducted following the protocol. Initially, Aβ_1–42_ peptide was reconstituted in sterile water to achieve a final concentration of 1 mM. The solution was then subjected to evaporation under a stream of high‐purity nitrogen gas at a temperature of 37°C for a duration of 30 min. Post‐evaporation, the peptide was allowed to incubate at ambient temperature for a period of 48 h to facilitate the formation of Aβ oligomers. Prior to application, the concentrated Aβ_1–42_ stock was appropriately diluted in sterile PBS to reach the specified concentrations.

### 2.10 Cell Culture and Transfection

Neuroblastoma SH‐SY5Y cells were purchased from the Cell Bank of the Chinese Academy of Sciences (Shanghai, China) and maintained in DMEM supplemented with 10% FBS (Hyclone). At 80% confluence, the SH‐SY5Y cells were treated with 10 μM Aβ_1–42_ (Sigma, St. Louis, MO, USA) for 24 h. Short hairpin RNA (shRNA) against SOX4, SNAP23, or IKKβ and the overexpression plasmids of TIP60 and SOX4 (pcDNA3.1‐TIP60, pcDNA3.1‐SOX4) were chemically synthesized by GenePharma. pcDNA3.1 empty vector acted as a negative control. Lipofectamine 3000 reagent (Invitrogen) was used for RNA transfections according to the instructions. The transfection efficiency was determined via RT‐qPCR.

### 2.11 Cell Counting Kit‐8 Assay

Cell viability was detected by cell counting kit‐8 (CCK‐8) assay (Dojindo, Japan). In short, after different treatments cells were harvested and washed with PBS solution. Then, 1 × 10^4^ of SH‐SY5Y cells were seeded into 96‐well plates, and 10 μL of CCK‐8 was added into each well for incubation 4 h at 37°C. Afterward, the 450 nm absorbance was measured using a microplate spectrophotometer (Bio‐Tek).

### 2.12 Flow Cytometry Assay

The apoptosis rate of SH‐5Y5Y cells was determined by the Annexin V/PI Apoptosis Detection Kit (BD Biosciences, CA, USA). In brief, SH‐SY5Y cells were collected and stained with binding buffer containing Annexin V‐FITC and PI in the dark for 15 min. Afterward, apoptosis was evaluated by flow cytometry (BD, NJ, USA), and data analysis was calculated by FlowJo software (FlowJo, USA).

### 2.13 Immunofluorescence Staining for LC3B + LAMP1 Staining

SH‐SY5Y cells grown on coverslips were fixed with 4% PFA and stained with the following the primary antibodies, including anti‐LAMP1 (ab25630, Abcam) and LC3B (#83506, Cell Signaling Technology) for overnight. The next day, the slides were incubated with the Alexa Fluor 488 conjugate secondary antibody (Cell Signaling Technology) and Alexa Fluor 594 conjugate antibody (ab150077, Abcam) for visualization. The nuclei were labeled with DAPI, and the photos were acquired under a fluorescence microscope (Leica, Japan). LC3B and LAMP1 double‐stained cells were quantified with the Image J software.

### 2.14 Lyso‐Tracker Red Staining

Lysosomal staining of SH‐SY5Y cells was incubated with Lyso‐Tracker Red (Thermo Fisher Scientific) for 30 min and washed 3 times with PBS solution. Finally, the cells were observed under a fluorescence microscope (Leica, Japan).

### 2.15 Co‐Immunoprecipitation Assay

SH‐SY5Y cells were lysed in IP lysis buffer containing a protease inhibitor cocktail and a phosphatase inhibitor cocktail (Beyotime, China). A small amount of protein was taken as the input control. Other parts of the lysates were immunoprecipitated with antibodies and Protein A/G beads (Invitrogen, USA) at 4°C to pull down. Finally, the eluted proteins were subjected to Western blot.

### 2.16 Dual Luciferase Assay

To confirm the binding relationship between SOX4 and the IKKβ promoter, the promoter sequences of IKKβ were cloned and inserted into pGL3basci vectors (Promega, WI, USA) for constructing recombinant luciferase vectors. SH‐SY5Y cells were co‐transfected with the recombinant luciferase vectors and pcDNA3.1‐SOX4 or sh‐SOX4 as well as their NCs (pcDNA3.1, shNC) by using lipofectamine 3000 reagent (Invitrogen). The relative luciferase activity by the Dual‐Luciferase Reporter System (Promega).

### 2.17 Chromatin Immunoprecipitation Assay

The chromatin immunoprecipitation (ChIP) assay was performed using the simple ChIP plus enzymatic chromatin IP kit (Cell Signaling Technology, USA) according to the manufacturer's protocols. Briefly, SH‐SY5Y cells were crossed‐linked with 1% formaldehyde and then quenched with glycine. Then, the DNA was sheared to 200–400 bp fragments by sonication. Finally, the lysate was immunoprecipitated with anti‐SOX4 (ab86809, Abcam) or IgG antibodies (ab172730, Abcam). Precipitated DNA was amplified and detected by RT‐qPCR.

### 2.18 Data Analysis

Data analysis was conducted using GraphPad Prism software (GraphPad, CA, USA) and presented as mean ± standard (SD) deviation of three independent experiments. Specifically, animal experiments were conducted for 12 times, and cellular assays were carried out on three separate occasions. Within each independent biological experiment, technical replicates were performed thrice to ensure the reliability and reproducibility of our findings. Prior to statistical analysis, all data sets were rigorously tested for normality using the Shapiro–Wilk test. Differences were made by the two‐tailed Student's *t‐test* between two groups or by one‐way ANOVA for comparisons in cases > 2 groups. Non‐normal data were analyzed using the Kruskal–Wallis *H* test. MWM tests were evaluated by repeated‐measures ANOVA followed by Tukey's post hoc tests of the between‐subject factor, while daily scores were compared using a *t*‐test with a significant set at *p* < 0.05.

## 3 Results

### 3.1 Gain‐Of‐Function of TIP60 Improves the Cognitive Ability of APP/PS1 Transgenic Mice

**FIGURE 1:**
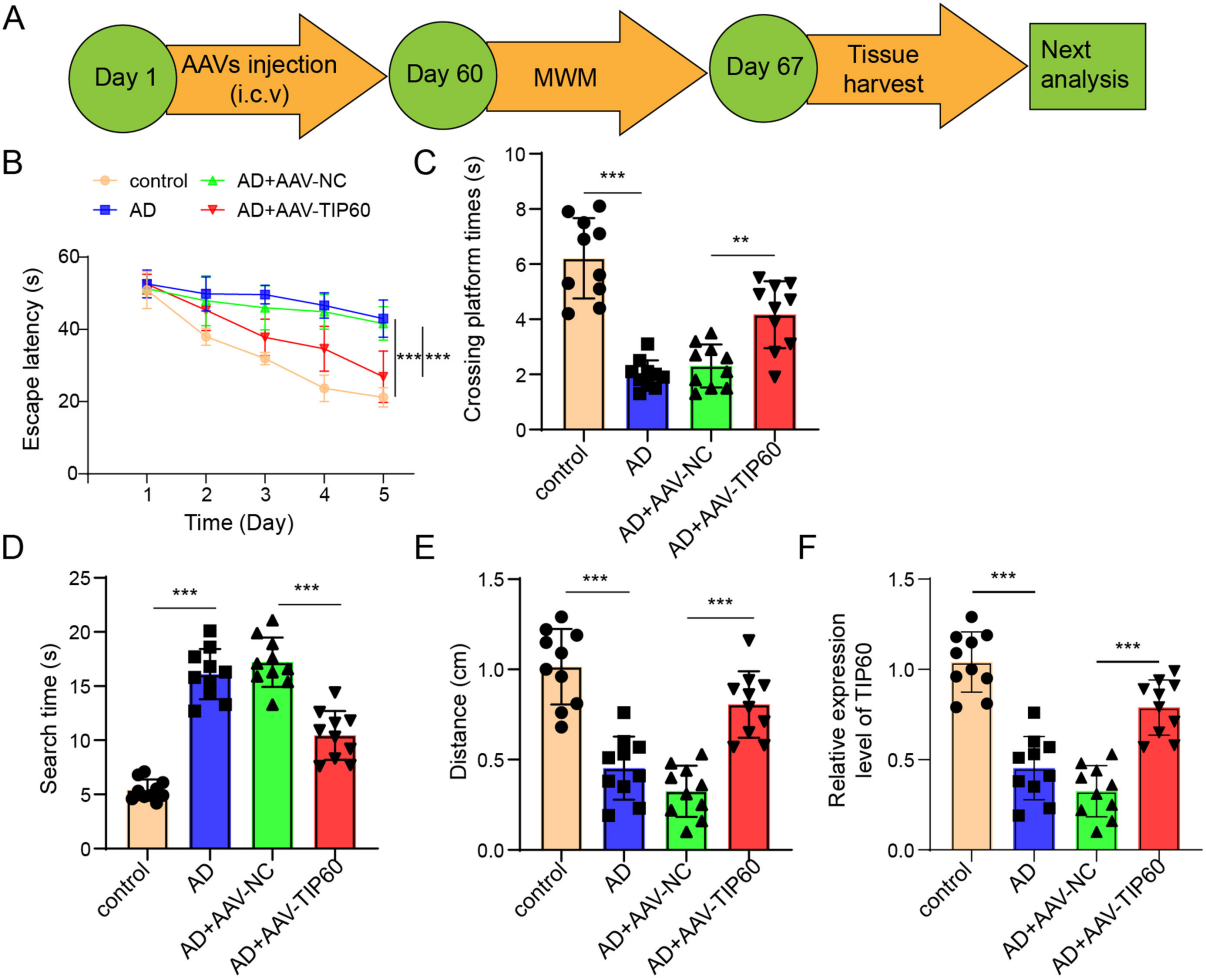
The effect of TIP60 on the cognitive ability in APP/PS1 transgenic mice. (A) Scheme for the in vivo experimental protocols in APP/PS1 mice. Mice received intracerebroventricular (i.c.v.) injection for 60 days until the beginning of the cognitive tests. From the 60th day of the experimental protocol, mice were subjected to MWM test. On the 67th day, the mice were euthanized to harvest tissues for further analysis. (B) The escape latency. (C) The number of times for mice crossing the platforms. (D) The target quadrant time. (E) The targeted distance. (F) RT‐ qPCR for TIP60 expression detection. **p < 0.01, ***p < 0.001.

APP/PS1 transgenic mice were used as the study subjects to investigate the potential therapeutic effect of TIP60 on cognition impairment in AD mice. The experimental procedure in APP/PS1 mice in vivo was presented in Figure 1A. After 2 months, the effect of TIP60 on cognitive ability was detected by MWM tests. Compared with control mice, AD mice or AD+AAV‐NC mice exhibited an increased escape latency (Figure 1B), fewer recorded times of crossing the platforms (Figure 1C), the longer search time (Figure 1D), and the shorter total moving distance (Figure 1E), while all these effects were improved after AAV‐TIP60 injection. RT‐qPCR data showed that TIP60 expression was decreased in hippocampus tissues of AD mice than that in the control group, while AAV‐TIP60 injection greatly enhanced TIP60 expression compared to AAV‐NC infection (Figure 1F). These data indicated that gain‐of‐function of TIP60 improved the cognitive ability in AD mice.

### 3.2 Overexpression of TIP60 Decreases Neurological Damage in APP/PS1 Transgenic Mice

**FIGURE 2:**
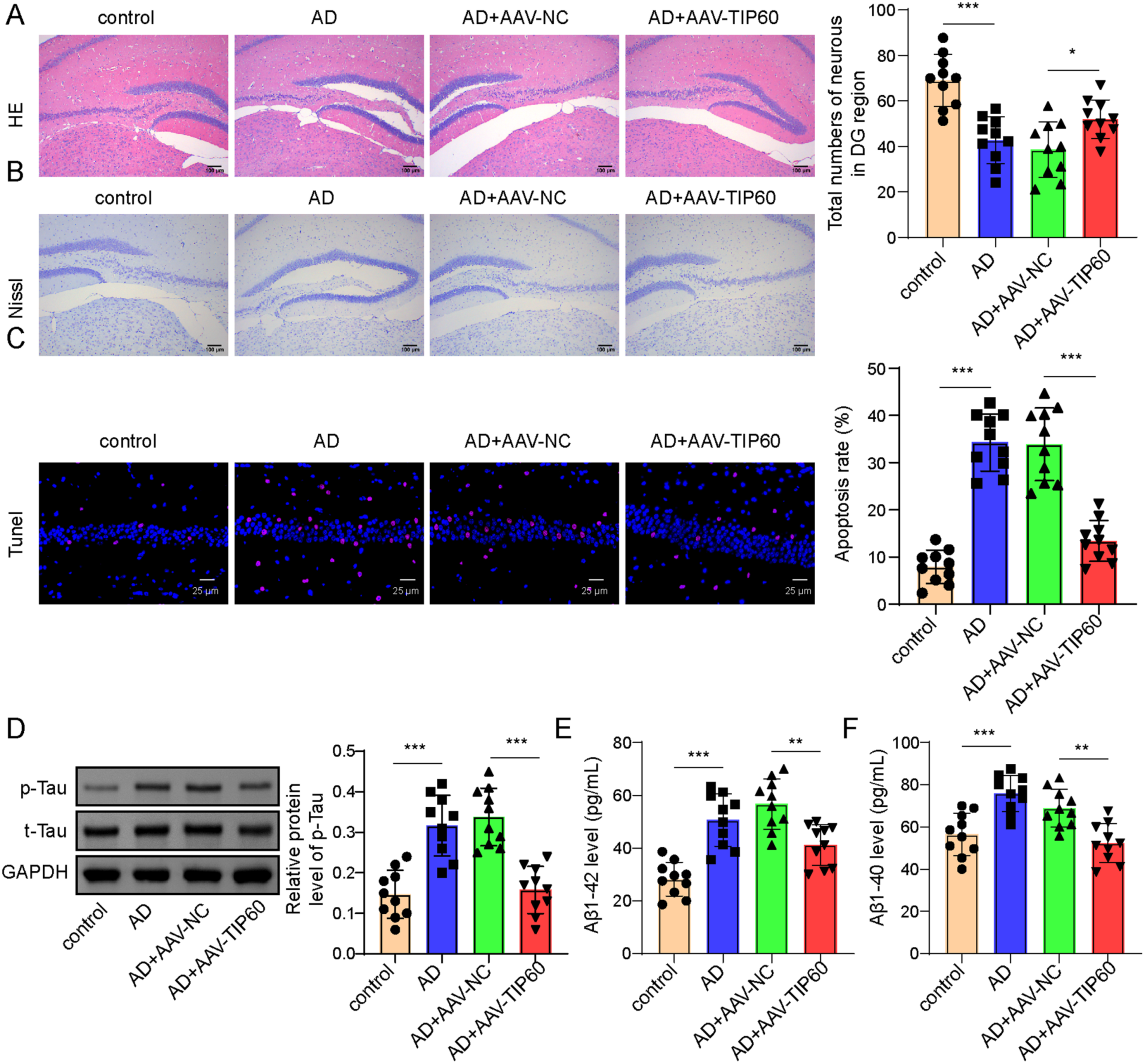
The effect of TIP60 on the neurological damage in APP/PS1 transgenic mice. (A, B) Representative pictures and quantitative analysis of HE and Nissl's staining in the DG region of hippocampus tissues. (C) Representative images and quantitative analysis of TUNEL staining. Green indicated TUNEL‐positive cells, red indicated NeuN, and blue indicated DAPI‐stained nuclei. (D) Western blot for p‐Tau (396) and t‐Tau detection. (E, F) ELISA for the measurement of Aβ40 and Aβ42 contents. *p < 0.05, **p < 0.01, ***p < 0.001.

To further evaluate the pathological effect of TIP60 overexpression on AD mice, HE and Nissl's staining were employed to access the neurological damage. As shown in Figure 2A,B, the AD group mice showed a disordered arrangement of neurons in the DG region of hippocampal tissue, slight changes in cell polarity, and a decrease in the number of neurons compared with the control group mice. However, TIP60 overexpression improved the morphology and arrangement of neurons and obviously increased the number of neurons. Moreover, the TUNEL assay confirmed that the neuronal apoptosis was strikingly elevated in hippocampal tissues from AD group mice, while this role could be dramatically overturned by TIP60 overexpression (Figure 2C). Western blot analysis illustrated that the level of p‐Tau was elevated in AD mice, while TIP60 overexpression remarkably diminished its elevation (Figure 2D). Moreover, ELISA analysis was conducted for the measurement of Aβ40 and Aβ42 contents. The results showed that both Aβ40 and Aβ42 were increased in AD group mice, while their levels could be restored upon TIP60 was overexpressed **(**Figure 2E,F **)**. These data indicated that overexpression of TIP60 decreased neurological damage and apoptosis in AD.

### 3.3 Upregulation of TIP60 Restores the Impairment of Autophagy‐Lysosomes Pathway

**FIGURE 3:**
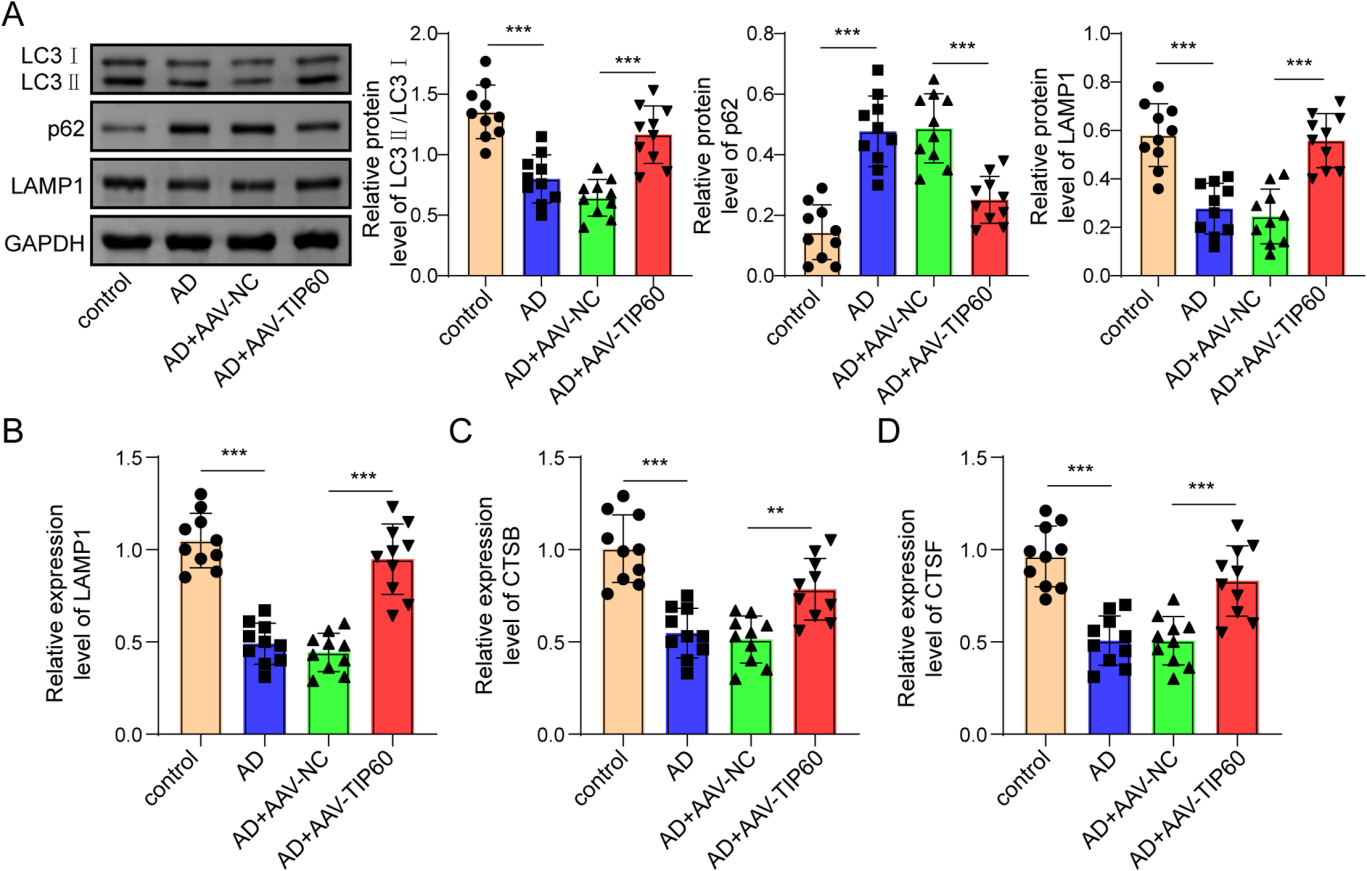
The effect of TIP60 on the impairment of the autophagy‐lysosomes pathway. (A) Western blot was performed to detect autophagy‐lysosomes‐related protein levels (LC3II/I, P62, and LAMP). (B) RT‐qPCR for the detection of lysosomal genes (LAMP1, CTSB, and CTSF). **p < 0.01, ***p < 0.001.

Autophagy‐lysosome is an important cellular process that cleans up the aggregated proteins and aging damage organelles thereby maintaining cellular homeostasis and protecting neurodegeneration [22]. In order to investigate whether TIP60 could affect the autophagy‐lysosomes pathway, we detected the expression levels of autophagy and lysosomal‐related genes/proteins. Western blot showed that compared with the control group, the levels of LC3II/I and LAMP1 in the hippocampus tissues of AD group mice was decreased, while p62 significantly was accumulated, and all these protein changes were reversed after AAV‐TIP60 injection (Figure 3A). Moreover, RT‐qPCR assay revealed that the mRNA expression of lysosomal related genes (LAMP1, CTSB, and CTSF) in the hippocampus tissues of AD group mice were decreased compared with the control group, and these effects were significantly reversed after TIP60 overexpression (Figure 3B). These data indicated that upregulation of TIP60 could restore the impairment of the autophagy‐lysosomes pathway in AD mice.

### 3.4 Overexpression of TIP60 Alleviates Neuron Damage Through Activating Autophagy‐Lysosomes Pathway

**FIGURE 4:**
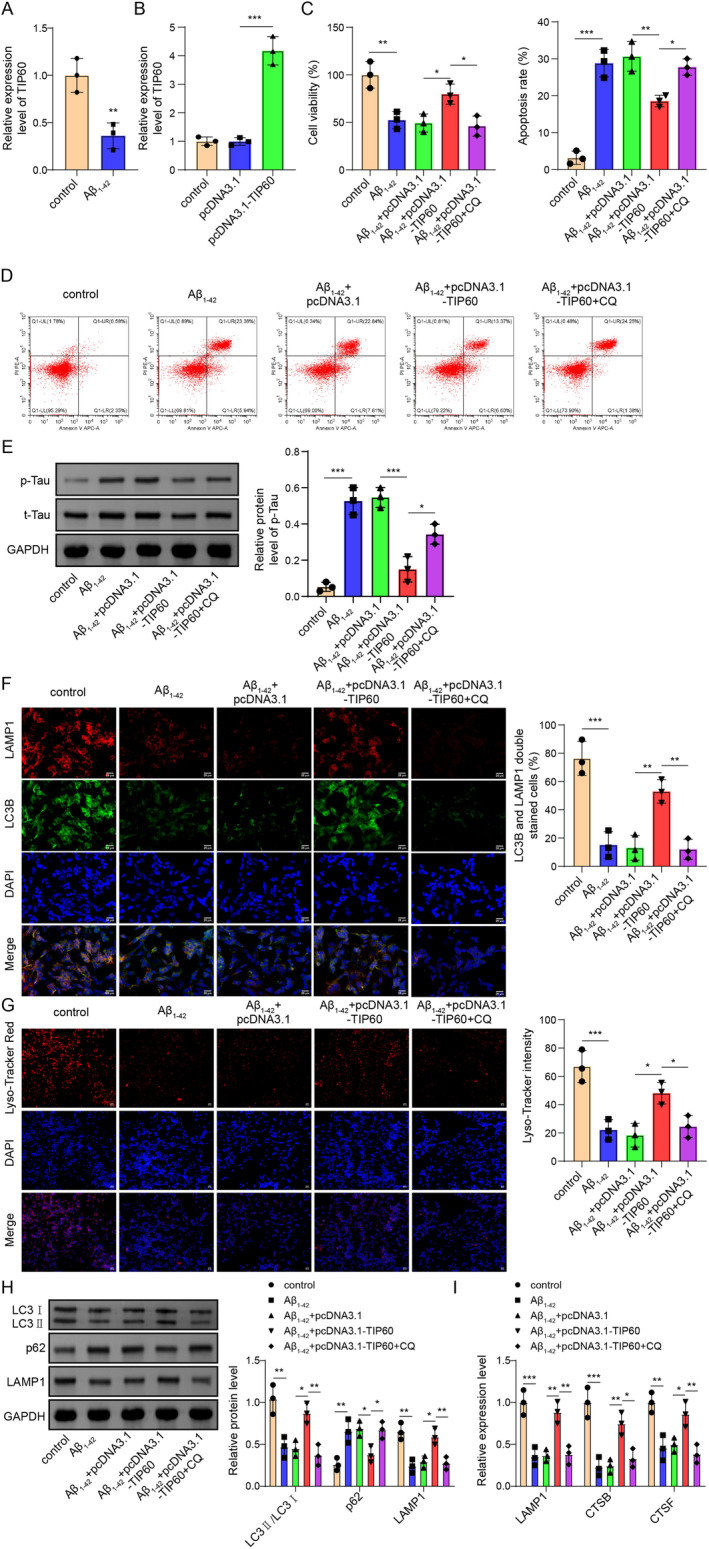
TIP60 alleviated neuron damage by activating autophagy‐lysosomes in SH‐SY5Y cells. (A) RT‐qPCR analysis of TIP60 after Aβ_1–42_ treatment. (B) RT‐qPCR analysis was performed to detect the transfection efficiency of pcDNA3.1‐TIP60. (C) SH‐SY5Y cell viability was determined by CCK‐8 assay. (D) The apoptotic rate was detected by flow cytometry. (E) Western blot for p‐Tau (Ser396) and t‐Tau detection. (F) Immunofluorescence staining for LC3B (green) + LAMP1 (red). (G) Lyso‐Tracker Red detected lysosomal function. (H) Western blot determined autolysosome‐related proteins (LC3II/I, P62, and LAMP1). (I) RT‐qPCR for the detection of lysosomal genes (LAMP1, CTSB, and CTSF). *p < 0.05, **p < 0.01, ***p < 0.001.

The SH‐SY5Y cells treated with Aβ_1–42_ were used as a cell model for AD. As shown in Figure 4A, Aβ_1–42_ treatment significantly decreased TIP60 mRNA expression level compared with control cells. Next, cells were transfected with pcDNA3.1 or pcDNA3.1‐TIP60, and the transfection efficiency of TIP60 was assessed by RT‐qPCR, which showed that TIP60 expression was up‐regulated after pcDNA3.1‐TIP60 transfection (Figure 4B). Then, the potential role of TIP60 on autophagosome‐lysosome in Aβ_1–42_ treated SH‐SY5Y cells was further examined. Results from the CCK‐8 and flow cytometry assay revealed that Aβ_1–42_ treatment reduced neuronal cell viability and increased apoptosis, while overexpression of TIP60 significantly alleviated these effects of Aβ_1–42_ treatment on neuronal proliferation and apoptosis (Figure 4C,D). Moreover, co‐treatment with CQ reversed the protective effects of TIP60 overexpression on neuronal proliferation and apoptosis (Figure 4C,D). Data from the Western blot assay showed that Aβ_1–42_ treatment increased the expressions of p‐Tau (396), while overexpression of TIP60 significantly reversed the expression trend. However, the effects of overexpression of TIP60 were significantly eliminated after CQ cotreatment (Figure 4E). Additionally, we performed immunofluorescence staining to check the co‐localization of LC3II and LAMP1 proteins. As shown in Figure 4F, Aβ_1–42_ treatment markedly reduced the expressions and colocalization of LCB and LAMP1, and the effect was markedly abolished after TIP60 overexpression, whereas the promoting role of TIP60 on colocalization of LCB and LAMP1 was further eliminated by CQ. Similarly, the Lyso‐Tracker Red assay showed the TIP60 overexpression significantly enhanced lysosomal function, while this effect was reversed upon CQ cotreatment (Figure 4G). Western blot detection disclosed that the levels of LC3II/I and LAMP1 were declined but p62 was elevated after Aβ_1–42_ treatment, and all these protein changes were greatly restored by TIP60 upregulation, and CQ co‐treatment could dramatically diminish these effects of TIP60 overexpression on these proteins (Figure 4H). Besides, Aβ_1–42_ treatment remarkably lightened the expressions of lysosomal associated genes (LAMP1, CTSB, and CTSF) in SH‐5Y5Y cells, and these roles could be weakened by TIP60 overexpression and further aggravated by CQ co‐treatment (Figure 4I). Taken together, the above findings proved that overexpression of TIP60 alleviated neuron damage by activating the autophagy‐lysosomes pathway in vitro.

### 3.5 TIP60 Facilitates the Interactions Between SNAP23 and STX17‐SNARE Complex to Induce Autophagosome‐Lysosome Fusion

**FIGURE 5:**
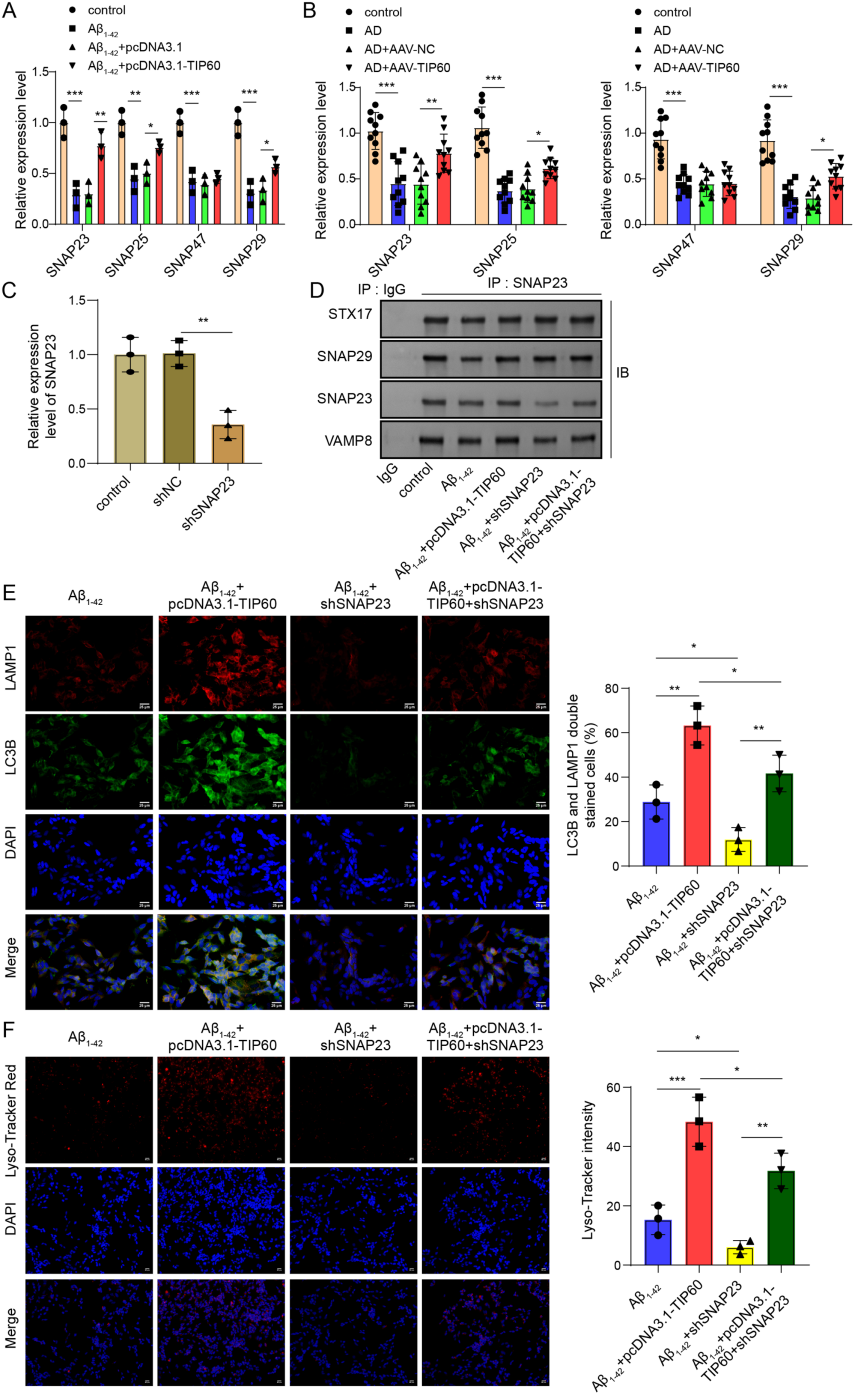
TIP60‐induced autophagosome‐lysosome fusion and SNAP23 and STX17‐SNARE complex interactions. (A) RT‐qPCR measured SNAP25, SNAP23, SNAP47, and SNAP29 expression in differently treated SH‐5Y5Y cells. (B) RT‐qPCR measured SNAP25, SNAP23, SNAP47, and SNAP29 expression in hippocampus tissues from each group of mice. (C) RT‐qPCR assessed the knockdown efficiency of SNAP23. (D) Co‐IP assay validated the interactions between SNAP23 and STX17‐SNAP29‐VAMP8 complex. (E) Immunofluorescence staining for LC3B + LAMP1 staining. (F) Lyso‐Tracker Red detected lysosomal function. *p < 0.05, **p < 0.01, ***p < 0.001.

The autophagy process involves membrane fusion, which mediates cell membrane substance transport and requires the interaction of SNARE family members to form a SNARE complex. Next, we detected the expression changes of SNAPs (including SNAP25, SNAP23, SNAP47, and SNAP29) in AD. The mRNA expression levels of SNAP25, SNAP23, SNAP47, and SNAP29 were all downregulated to varying degrees both in hippocampal tissue of AD mice and Aβ_1–42_‐treated SH‐5Y5Y cells. After overexpression of TIP60, the expressions of SNAP25, SNAP23, and SNAP29 were increased except SNAP47. Among them, SNAP23 was identified as the most upregulated gene (Figure 5A,B). Previous studies have shown that SNAP23 could interact with the autophagosome STX17‐SNAP29‐VAMP8 complex to form autophagosomes and participate in the fusion between autophagosomes and lysosomes [19]. Therefore, we further explored whether TIP60 participated in the autophagy‐lysosome fusion process through SNAP23 by silencing SNAP23 expression in SH‐SY5Y cells. As shown in Figure 5C, the knockdown efficiency of SNAP23 was confirmed by RT‐qPCR **(**Figure 5D **)**. Co‐Immunoprecipitation (Co‐IP) revealed the interactions between SNAP23 and SNARE complex. Compared to the control group, Aβ_1–42_ treatment reduced interaction between SANAP23 and STX17‐SNAP29‐VAMP8 complex, while this phenomenon was significantly reversed by TIP60 overexpression and enforced by SNAP23 knockdown. It was worth noting that the promoting effect of TIP60 overexpression on the formation of SNAP23‐STX17‐SNAP29‐VAMP8 complex was significantly eliminated after SNAP23 silencing (Figure 5D), suggesting that TIP60 relied on SNAP23 to promote the formation of SNAP23‐STX17‐SNAP29‐VAMP8 complex. Moreover, it was also observed that TIP60 overexpression significantly increased the expression and co‐localization of LC3II and LAMP1 and improved lysosomal function compared with the Aβ_1–42_ treatment group. In contrast, silencing of SNAP23 had the opposite effect as TIP60 overexpression and significantly reversed the regulatory effect of TIP60 overexpression on autophagy‐lysosomal fusion (Figure 5E,F).

### 3.6 IKKβ Is Essential for the Translocation of SNAP23 to STX17‐Contained Autophagosomes Mediated by TIP60

**FIGURE 6:**
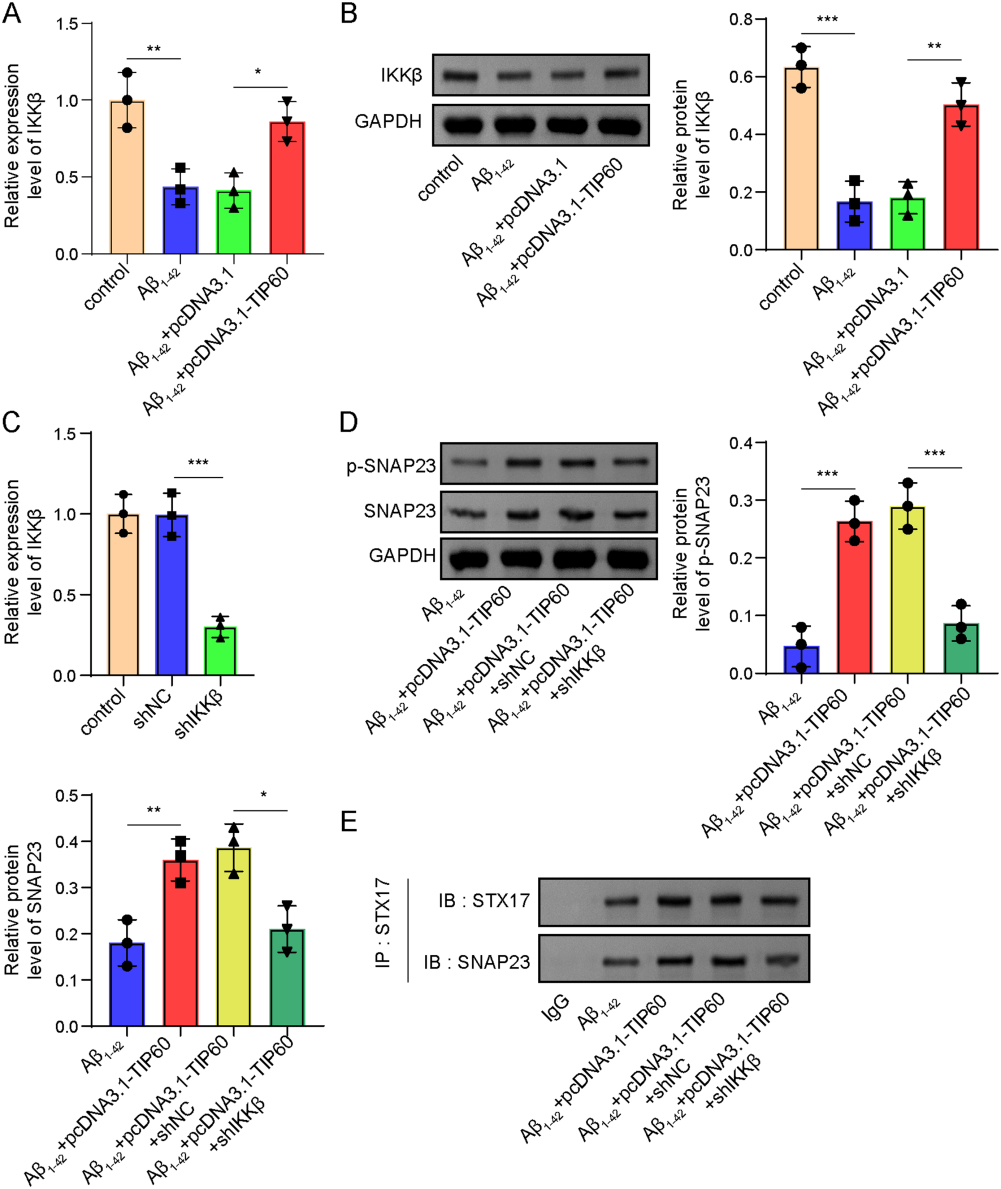
IKKβ participated in the translocation of SNAP23 to STX17‐contained autophagosomes mediated by TIP60. (A, B) RT‐qPCR and Western blot measured IKKβ expression. (C) RT‐qPCR assessed the knockdown efficiency of IKKβ. (D) Western blot evaluated the protein level of SNAP23. (E) Co‐IP examined the interaction between SNAP23 and STX17. *p < 0.05, **p < 0.01, ***p < 0.001.

We further investigated the underlying mechanism of TIP60‐mediated SNAP23 translocation to STX17‐contained autophagosomes. In SH‐SY5Y cells, Aβ_1–42_ treatment reduced IKKβ expression, while overexpression of TIP60 further restored IKKβ expression (Figure 6A,B). In addition, the SH‐SY5Y cell line with silenced IKKβ was constructed, and the silencing efficiency was confirmed by RT‐qPCR analysis (Figure 6C). Western blot assay showed that enforced expression of TIP60 increased the protein level of SNAP23, and this effect was eliminated after IKKβ silence (Figure 6D). Furthermore, Co‐IP assay validated that overexpression of TIP60 promoted the binding relationship between STX17 and SNAP23, and this change was eliminated after IKKβ depletion (Figure 6E). Together, these results suggested that IKKβ participated in the formation of SNAP23‐STX17‐contained autophagosomes mediated by TIP60.

### 3.7 TIP60 Accelerates IKKβ Transcription via Enhancing SOX Acetylation

**FIGURE 7:**
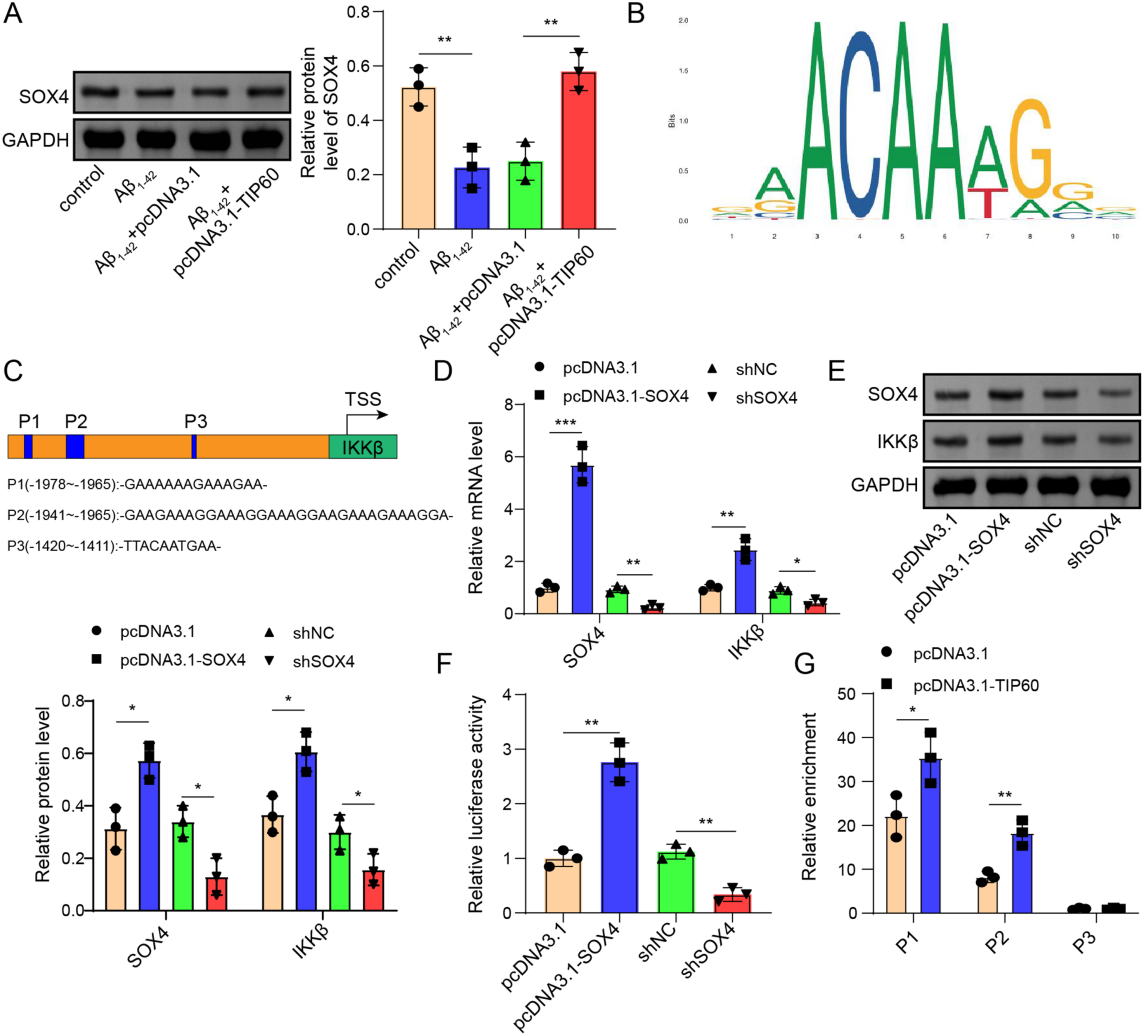
TIP60 accelerated IKKβ transcription via enhancing SOX acetylation. (A) Western blot tested the protein level of SOX4. (B, C) Schematic representation of the SOX4 motif and the binding sites of SOX4 to the IKKβ promoter. (D, E) RT‐qPCR and Western blot detected the expressions of SOX4 and IKKβ. (F) A dual luciferase assay measured the binding relationship between SOX4 and the IKKβ promoter. (G) ChIP verified the direct interaction between SOX4 and the IKKβ promoter (P1, P2, P3). *p < 0.05, **p < 0.01, ***p < 0.001.

Tip60, recognized as a lysine HAT, selectively identifies and catalyzes the acetylation of specific lysine residues on core histones, such as H2AK5ac, H3K9ac, H3K14ac, H4K8ac, H4K12ac, and H4K16ac [23, 24, 25, 26]. Western blot results demonstrated that the total Ac‐Lys, H2AK5ac, H3K9ac, H3K14ac, H4K8ac, H4K12ac, and H4K16ac proteins were significantly reduced in AD mice and SH‐5Y5Y cell models, and these changes were significantly alleviated after overexpression of TIP60 (Figure S1A,B). This indicates that TIP60 plays a crucial role in regulating histone acetylation, which in turn influences the expression of genes involved in AD pathology. TIP60 has also been confirmed to participate in chromatin remodeling in the promoter region of its target gene by influencing the acetylation of SOX4 at lysine 95 [23]. A Co‐IP assay was conducted to analyze the physical interaction between SOX and TIP60, or Ac‐Lys. The results, depicted in Figure S1C,D demonstrated that the interaction between SOX4 and TIP60 or Ac‐Lys was inhibited in AD mice and cell models but enhanced after overexpression of TIP60. Western blot analysis revealed that the expression of SOX4 protein was downregulated in cells treated with Aβ_1–42_, but SOX4 protein levels were restored after overexpression of TIP60 (Figure 7A). After JASPAR prediction, the transcriptional sites (P1, P2, and P3) of SOX4 on the IKKβ promoter were found, and the motif of SOX4 and the potential binding relationship between SOX4 and the IKKβ promoter were described in Figure 7B,C. Next, RT‐qPCR and Western blot assays showed that pcDNA3.1‐SOX4 transcription greatly elevated SOX4 and IKKβ levels, and sh‐SOX4 transfection significantly repressed SOX4 and IKKβ levels (Figure 7D,E). Later, dual luciferase assays showed that overexpression of SOX4 resulted in a significant increase in IKKβ promoter activity. In contrast, silencing of SOX4 exhibited an opposite effect (Figure 7F). To validate the direct binding relationship between SOX4 and the IKKβ promoter region, a ChIP assay was confirmed and found that SOX4 could bind to the P1 and P2 regions but not the P3 region (Figure 7G). These results suggested that TIP60 accelerated IKKβ transcription via enhancing SOX4 acetylation.

### 3.8 Inhibition of SOX4/IKKβ Signaling Diminish the Protective Roles of TIP60 Upregulation in Aβ_1–42_‐Induced Neuron Damage

**FIGURE 8:**
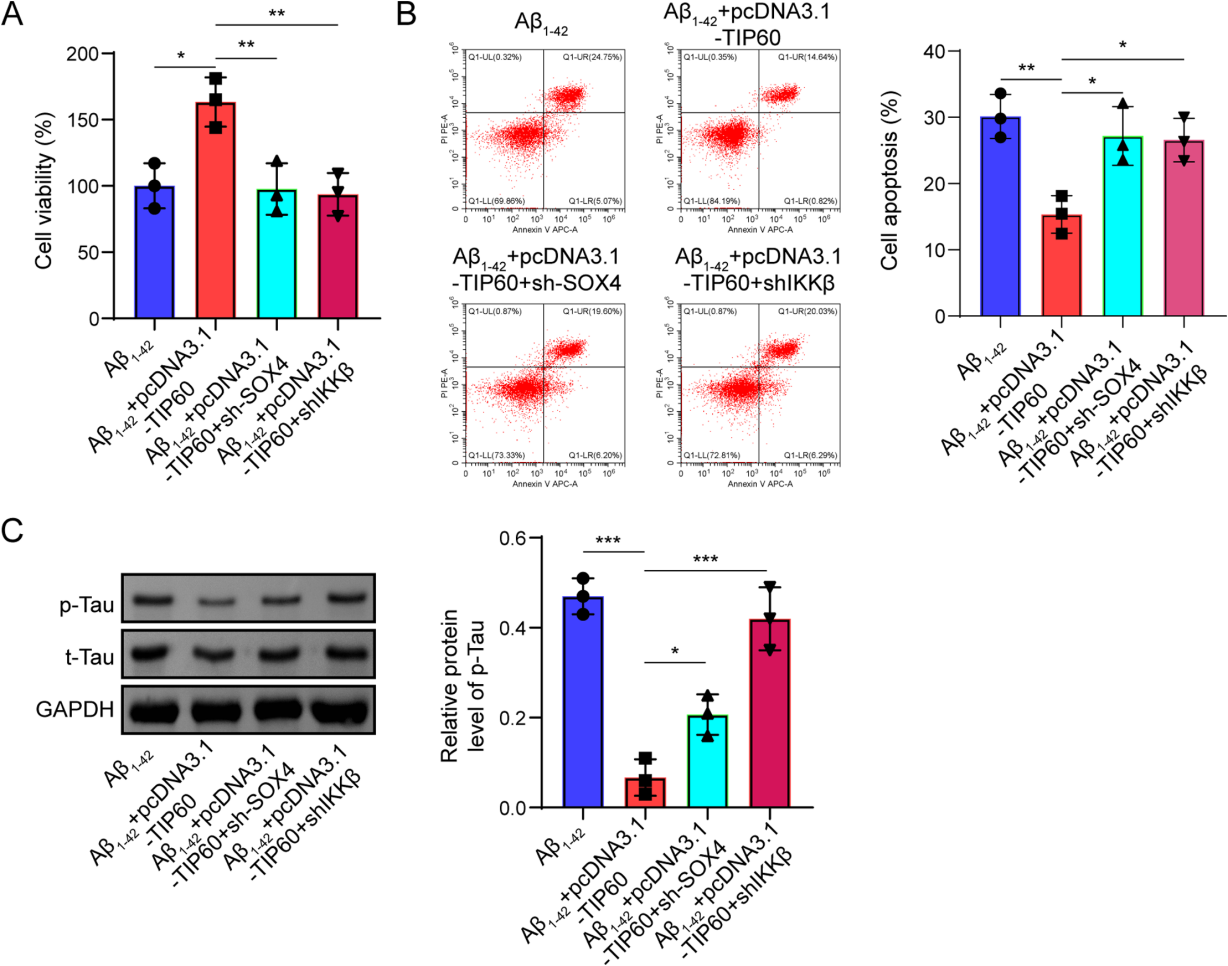
Inhibition of SOX4/IKKβ signaling diminished the protective roles of TIP60 upregulation in Aβ_1–42_‐induced neuron damage. (A) CCK‐8 detected cell viability. (B) Flow cytometry evaluated cell apoptosis. (C) Western blot detected the protein levels of p‐Tau and t‐Tau. *p < 0.05, **p < 0.01, ***p < 0.001.

In order to verify the effects of SOX4/IKKβ on the neuroprotective role of TIP60, we utilized shRNA targeting SOX4 or IKKβ to inactivate SOX4/IKKβ cascades. Results from the CCK‐8 assay and flow cytometry assay revealed that the elevation of TIP60 promoted cell proliferation and restrained apoptosis in Aβ_1–42_‐induced SH‐5Y5Y cells, while these roles were dramatically reversed upon SOX4 or IKKβ was silenced (Figure 8A,B). Furthermore, Western blot assay revealed that enforced expression of TIP60 remarkably decreased the protein level of p‐Tau (396), while silencing of SOX4 or IKKβ reversed the protein trend (Figure 8C). These outcomes implied that inactivation of SOX4/IKKβ signaling greatly diminished the protective roles of TIP60 on Aβ_1–42_‐induced neuron injury.

### 3.9 The Effects of TIP60 Overexpression Acted on Autophagy‐Lysosomes Pathway Was Depended on SOX4/IKKβ Signaling Pathway

**FIGURE 9:**
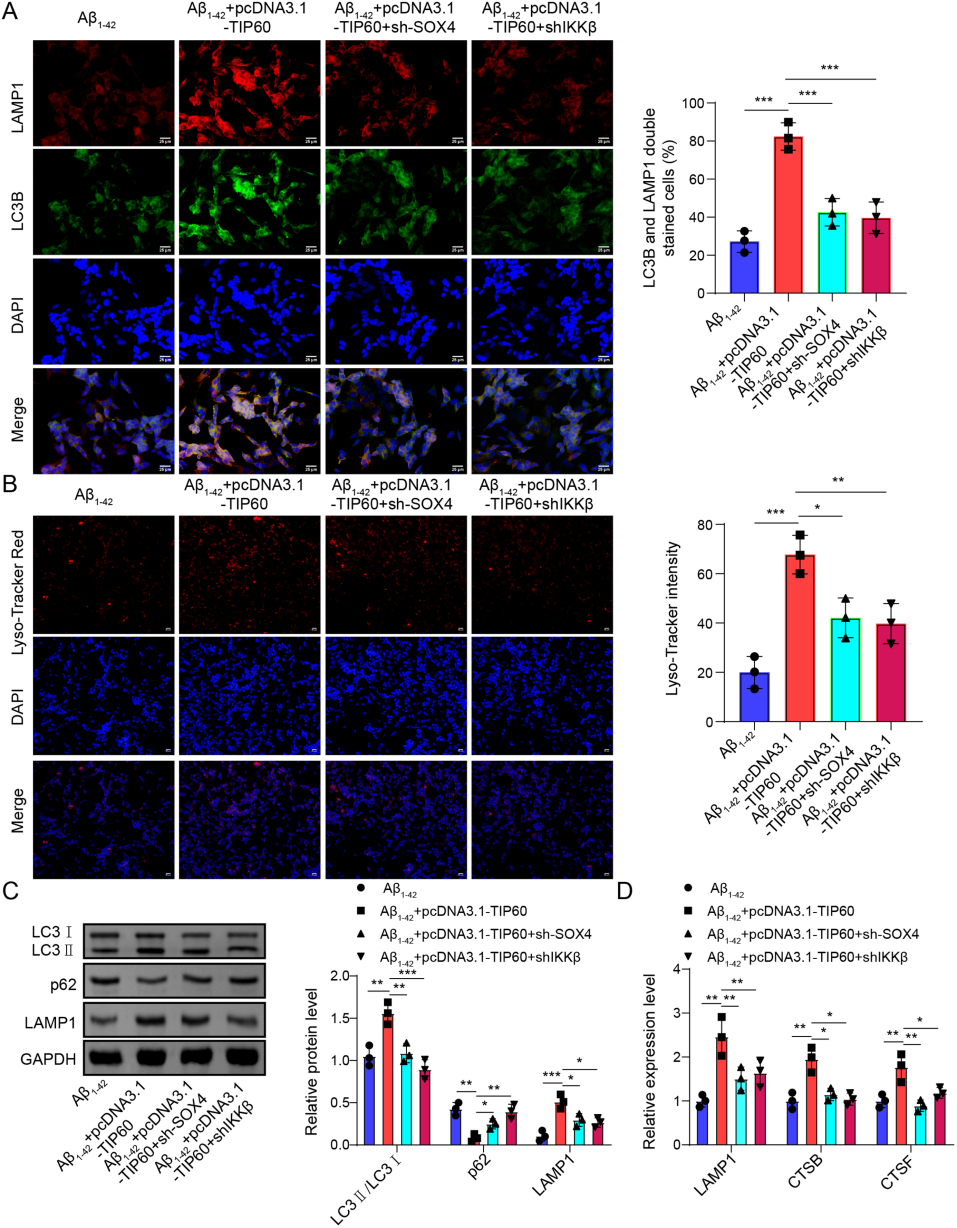
The effects of TIP60 overexpression on autophagy‐lysosomes pathway through SOX4/IKKβ signaling. (A) Immunofluorescence staining for LC3B (green) + LAMP1 (red). (B) Lyso‐Tracker Red detected lysosomal function. (C) Western blot quantified the autolysosome‐related proteins (LC3II/I, P62, LAMP1). (D) RT‐qPCR determined expressions of lysosomal genes (LAMP1, CTSB, CTSF). *p < 0.05, **p < 0.01, ***p < 0.001.

We further investigated the effects of SOX4/IKKβ on TIP60‐meidated effects on the autophagy‐lysosomes pathway. As shown in Figure 9A, overexpression of TIP60 greatly increased the colocalization of LC3B and LAMP1 and lysosome function, while these effects could be reversed by SOX4 or IKKβ knockdown **(**Figure 9B **)**. Western blot assay illustrated that loss of SOX4 or IKKβ remarkably lightened the promoting roles on LC3II/I, LAMP1, and the inhibitory role on p62 mediated by TIP60 overexpression **(**Figure 9C **)**. Similarly, the elevation of lysosomal associated genes (LAMP1, CTSB, and CTSF) mRNA caused by TIP60 overexpression in Aβ_1–42_‐induced SH‐5Y5Y cells was also diminished upon SOX4 or IKKβ downregulation **(**Figure 9D **)**. Taken together, these data showed that overexpression of TIP60 acted on the autophagy‐lysosomes pathway via regulating SOX4/IKKβ signaling.

## 4 Discussion

The pathological features of AD mainly include diffuse plaques formed by extracellular Aβ deposits and intracellular neurofibrillary tangles (NFT) formed by phosphorylated tau protein aggregation [27]. Numerous studies have shown that there are certain autophagy regulatory disorders in the brain lesions of AD patients, mainly due to the disruption of substrate protein hydrolysis in lysosomes [28, 29]. Therefore, autophagic modulation may be a key target for future AD therapeutics. In this study, we demonstrated that TIP60 promoted IKKβ transcription by acetylating SOX4, thereby increasing SNAP23‐mediated autophagy‐lysosome fusion and alleviating the AD progression.

APP/PS1 dual transgenic mice are AD models designed based on the etiology of AD and are widely used in AD research. Previously, it has been revealed that early exposure to Aβ_1–42_ disrupts the TIP60 HAT/HADC2 balance during the early neurodegenerative stage, while the increase in TIP60 HAT level can significantly improve AD pathology, including Aβ plaque accumulation, neuronal cell death, cognitive deficits, and reduced lifespan, preventing Aβ_1–42_‐induced transcriptomic changes [12]. Similarly, TIP60 expression effectively alleviates the control of Aβ peptide over synaptic and neuronal function genes, thereby alleviating AD [30]. Here, the present study revealed that AD mice exhibited pronounced cognitive impairment, neuronal damage, and autophagic lysosomal dysfunction, along with a significant increase in Aβ_1–42_ and Aβ_1–40_ levels and accumulation of phosphorylated tau protein; however, all these changes could be improved after TIP60 overexpression, indicating that TIP60 plays a protective role in AD pathology. Besides, it has been reported that TIP60 triggers acetylation of ULK1 in a GSK3β‐dependent manner to activate autophagy, promoting cells resistance to endoplasmic reticulum stress [31]. TIP60 also plays as an essential regulator of autophagy‐associated Beclin1 to involve in cell autophagy, G2/M cycle arrest, and survival [32]. It could be further confirmed our findings by which TIP60 functioned as an autophagy inducer to improve autophagic‐lysosomal disorder during AD. In addition, histone acetylation of chromatin is important for transcriptional responses in neurons that promote neuroplasticity and cognitive ability. TIP60 HAT activity is involved in the transcriptional regulation of neuronal genes and synaptic plasticity and has been implicated in AD [33]. Specific depletion of TIP60 HAT activity in the nervous system facilitates APP‐mediated lethality and neuronal apoptosis in the central nervous system of a transgenic AD fly model [10]. Also, loss of TIP60 in the nervous system leads to axonopathy and transport defects [34]. Studies have shown that the significant reduction in the number of brain neurons is an important pathophysiological change in AD, with the most severe decrease occurring in the basal forebrain and hippocampus, while apoptosis is a critical factor for the loss of neurons [35]. Our current research demonstrated that overexpression of TIP60 alleviated neuron damage and apoptosis, as evidenced by a decreased number of TUNEL‐positive neurons and an increased number of NeuN‐positive neurons in APP/PS1 mice, as well as an increase in cell viability and a decrease in apoptotic cells in Aβ_1–42_ treated neurons, whereas all of the protective roles of TIP60 were diminished upon autophagy‐lysosomes were inhibited. Collectively, our result demonstrated for the first time that TIP60 plays as a critical protector of autophagy‐lysosomes to prevent neurons from Aβ_1–42_‐induced cell damage and cognitive dysregulation.

In autophagosomes, SNAP23 binds to and regulates autophagosome lysosome fusion mediated by the STX17‐SNARE complex. Knockout of SNAP23 impaired the assembly of STX4‐driven basolateral exocytotic SNARE complex and STX17‐driven SNARE complex, resulting in reduction of autophagosome formation, thus protecting pancreatitis progression [19]. Kunii et al.'s study has shown that SNAP23 is closely related to various neuronal events, and specific knockout SNAP23 mice results in severe brain dysplasia by blocking the formation of the SNAP23‐VAMP8‐Syntaxin1B complex, which is a critical step in the formation of apical junction complexes and radial glial cell polarization [36]. Moreover, melatonin reverses Aβ_1–42_‐induced neurotoxicity and memory impairment by increasing the levels of presynaptic proteins (Synaptophysin and SNAP25) and postsynaptic proteins (PSD95 and SNAP23) [37]. However, the correlation between SNAP23 and autophagy‐lysosomes in the AD process has not been reported. Here, we first observed that the formation of the SNAP23‐STX17‐SNAP29‐VAMP8 complex was driven by TIP60 overexpression, which suggested the exact acting pathway of TIP60 in inducing autophagosome‐lysosome fusion during the AD process.

IKKβ is the core subunit in the IKK complex, and its kinase activity regulates inflammation, apoptosis, autophagy, and metabolism through non‐NF‐κB‐dependent mechanisms by phosphorylating target proteins such as SNAP23 and β‐catenin [38, 39]. Blocking IKKβ in bone marrow cells not only inhibited the activation of inflammatory reactions but also reduced the level of Aβ in the brains of AD mice [40]. Therefore, IKKβ may serve as an important target to alleviate neuronal degeneration and slow down the development of AD. Consistently, we found that Aβ_1–42_ treatment reduced IKKβ expression, while this effect was reversed by overexpression of TIP60. The membrane fusion in autophagy is mainly completed by the SNARE complex, and the assembly of the SNARE complex is determined by SNARE regulators and post‐translational modification (i.e., acylation and phosphorylation) [41]. Studies have demonstrated IKKβ phosphorylation of SNAP23 promoted its translocation from the inherent plasma membrane to autophagosomes [39]. In this study, we discovered that overexpression of TIP60 increased the protein level of SNAP23 and the binding between STX17 and SNAP23, while this effect was eliminated after IKKβ silence, demonstrating that IKKβ was essential for the translocation of SNAP23‐STX17‐contained autophagosomes mediated by TIP60. Importantly, our data further illuminated that IKKβ knockdown reversed the protective roles of TIP60 overexpression on neuron damage and the autophagy‐lysosomes pathway.

Previous studies have shown that during myocyte differentiation, TIP60 acetylated the SOX4 Lys95 site, promoting SOX4 recruitment to downstream gene promoter regions and increasing its transcription [23]. As a lysine HAT, it specifically recognizes and catalyzes the acetylation of lysine residues on core histones. Here, the total Ac‐Lys, H2AK5ac, H3K9ac, H3K14ac, H4K8ac, H4K12ac, and H4K16ac proteins were significantly reduced in AD mice and SH‐5Y5Y cell models, and these changes were significantly alleviated after overexpression of TIP60. Moreover, the interaction between SOX4 and TIP60, or Ac‐Lys, was inhibited in AD mice and cell models but enhanced after overexpression of TIP60, indicating that TIP60 exhibited acetyltransferase activity in both AD mice and cell models. In addition, our present work verified that TIP60 accelerated IKKβ transcription via enhancing SOX4 acetylation. Under physiological conditions, SOX4 protein plays a regulatory role in the differentiation of glial and neurons, as well as the formation of Schwann cell myelin sheath. After the differentiation and maturation of glial and neuronal cells, its expression gradually decreases [42, 43]. A previous study has revealed that miR‐31‐5p inactivates mTORC1 by directly targeting SOX4 to affect apoptosis and autophagy of chondrocytes, suggesting that SOX4 may play a protective role in the progression of osteoarthritis [44]. Moreover, dexmedetomidine reduces sevoflurane‐induced neurotoxicity by upregulating the expression level of SOX4 [45]. Our study demonstrated for the first time that SOX4 knockdown reversed the effects of TIP60 overexpression on neuroprotective roles and autophagy‐lysosome fusion, which elucidated the precise mechanism of TIP60‐SOX4‐IKKβ in autophagy‐lysosome fusion and neuronal damage.

## 5 Conclusion

In conclusion, the key findings of the present study suggested that TIP60 served a protective effect on nerve injury in AD by activating the IKKβ/SNAP23 axis‐mediated autophagosome‐lysosome fusion pathway. This provides new insights into potential therapeutic targets for AD treatment. However, more research is needed to fully understand the mechanisms involved in this pathway and to test potential therapeutic interventions. Additionally, the specific role of TIP60 in these processes requires further investigation.

## Author Contributions

Wei Wang conceived and designed the research. Jun Min, Qinghua Luo, and Xunhu Gu performed the research and acquired the data. Min Li, Wei Wang, and Xu Liu analyzed and interpreted the data. All authors were involved in drafting and revising the manuscript.

## Ethics Statement

This animal study was approved by the Ethics Committee of The Second Affiliated Hospital of Nanchang University. All the animal procedures were performed in accordance with the ARRIVE guidelines.

## Consent

The authors have nothing to report.

## Conflicts of Interest

The authors declare no conflicts of interest.

## Supporting information


Figure S1.

Figure S2.

## Data Availability

All data generated or analyzed are included in this article. Further inquiries can be directed to the corresponding author.
