# Supplementary figures and images for: Lysine Acetyltransferase TIP60 Restricts Nerve Injury by Activating IKKβ/SNAP23 Axis‐Mediated Autophagosome‐Lysosome Fusion in Alzheimer's Disease
Source: CNS Neurosci Ther. 2024 Nov 5;30(11):e70095. doi: 10.1111/cns.70095 (PMC11537769; doi:10.1111/cns.70095)

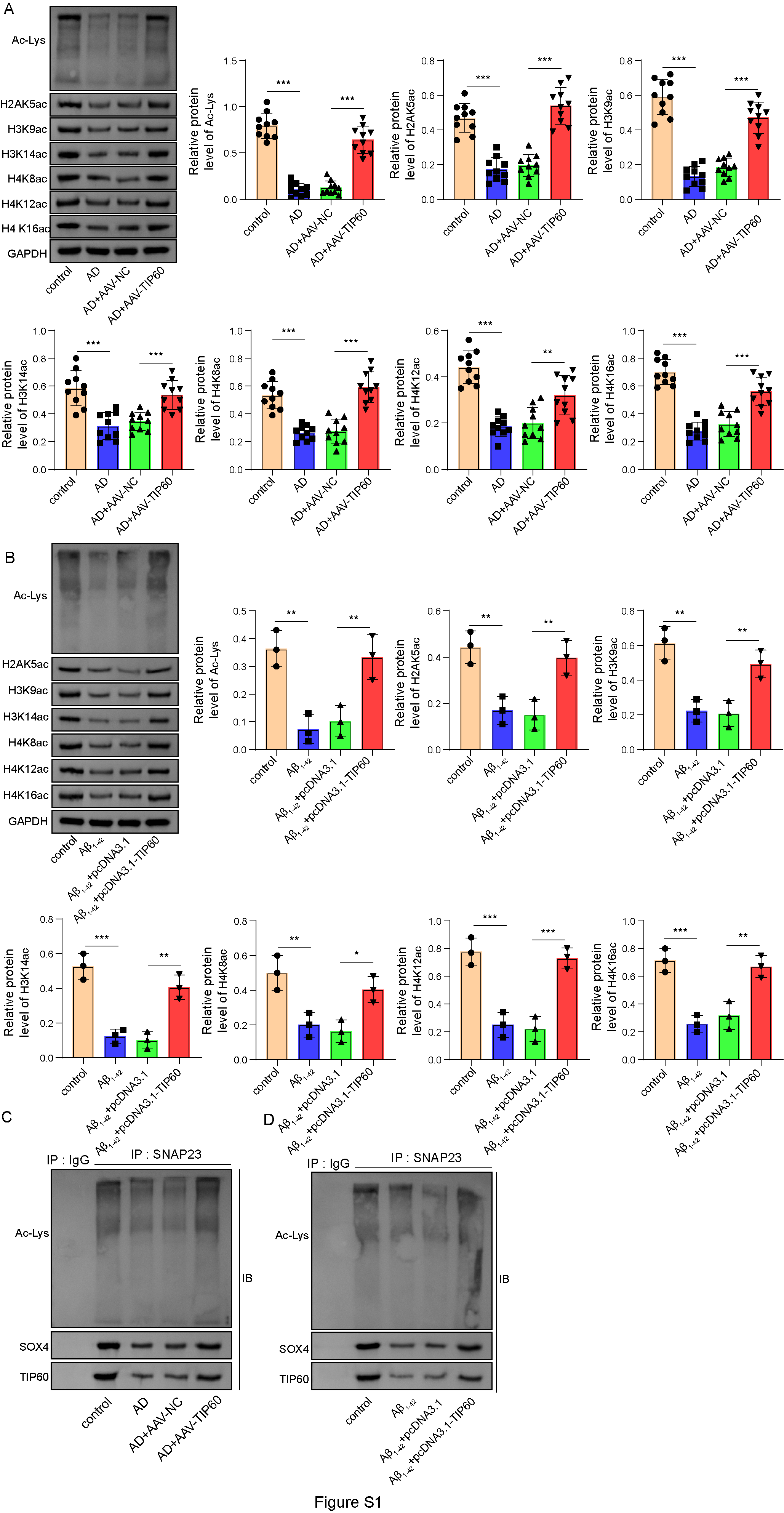

Supplement: Supplementary file 1 — Figure S1. [file CNS-30-e70095-s001.tif]

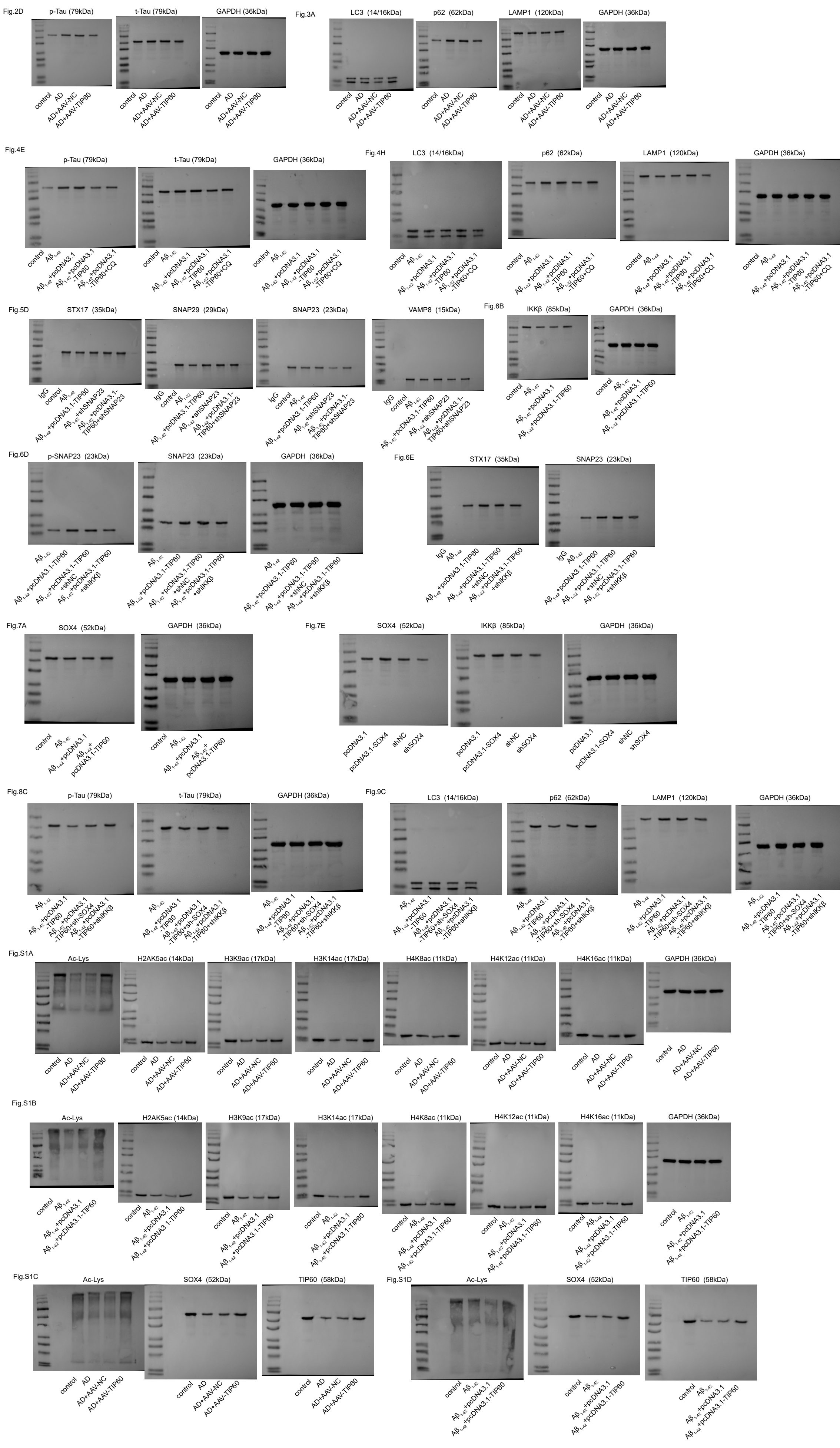

Supplement: Supplementary file 2 — Figure S2. [file CNS-30-e70095-s002.pdf]
